# Pharmacokinetic Variability Drives Palbociclib-Induced Neutropenia in Metastatic Breast Cancer Patients: Drug–Drug Interactions Are the Usual Suspects

**DOI:** 10.3390/pharmaceutics14040841

**Published:** 2022-04-11

**Authors:** Fanny Leenhardt, Frédéric Fiteni, Ludovic Gauthier, Marie Alexandre, Séverine Guiu, Nelly Firmin, Stéphane Pouderoux, Marie Viala, Gerald Lossaint, Chloé Gautier, Caroline Mollevi, Matthieu Gracia, Celine Gongora, Litaty Mbatchi, Alexandre Evrard, William Jacot

**Affiliations:** 1 Institut du Cancer de Montpellier, Université de Montpellier, 208 rue des Apothicaires, 34298 Montpellier, France; ludovic.gauthier@icm.unicancer.fr (L.G.); marie.alexandre@icm.unicancer.fr (M.A.); severine.guiu@icm.unicancer.fr (S.G.); nelly.firmin@icm.unicancer.fr (N.F.); stephane.pouderoux@icm.unicancer.fr (S.P.); marie.viala@icm.unicancer.fr (M.V.); gerald.lossaint@icm.unicancer.fr (G.L.); chloe.gautier@icm.unicancer.fr (C.G.); caroline.mollevi@icm.unicancer.fr (C.M.); william.jacot@icm.unicancer.fr (W.J.); 2 Institut de Recherche en Cancérologie de Montpellier, INSERM U1194, Université de Montpellier, 34090 Montpellier, France; matthieugracia@hotmail.fr (M.G.); celine.gongora@inserm.fr (C.G.); litaty.mbatchi@umontpellier.fr (L.M.); alexandre.evrard@umontpellier.fr (A.E.); 3 Laboratoire de Pharmacocinétique, Faculté de Pharmacie, Université de Montpellier, 34090 Montpellier, France; 4 Service d’Oncologie Médicale, CHU de Nîmes, CEDEX 9, 930029 Nîmes, France; frederic.fiteni@chu-nimes.fr; 5 Institut Desbrest d’Epidemiologyie et de Santé Publique, INSERM UMR 1302, Université de Montpellier, 34090 Montpellier, France

**Keywords:** clinical trial, oncology breast cancer, therapeutic drug monitoring, drug–drug interaction

## Abstract

Palbociclib is a good candidate for therapeutic drug monitoring (TDM) due to its narrow therapeutic range and frequency of toxicities, particularly high-grade neutropenia. In this prospective, bicentric clinical trial, we evaluated the palbociclib exposure–toxicity relationship and determined the relevant sources of palbociclib pharmacokinetic variability, including drug–drug interactions (DDI). We followed 58 patients (mean age: 62.9 years) for 1 year. The geometric median of palbociclib plasma trough concentration (C_trough_) was 74.1 ng/mL. Neutropenia occurred in 70.7% of patients (high grade in 67.2% of patients). High-grade neutropenia occurrence during the first two palbociclib cycles was higher in patients with lower neutrophil count at initiation (*p* = 0.002). Palbociclib plasma C_trough_ was correlated with high-grade neutropenia occurrence during the first two cycles (*p* = 0.024, OR 5.51). Co-treatment with agents that may interfere with palbociclib PK significantly influenced palbociclib C_trough_ (*p* < 0.05). CYP3A4/P-glycoprotein inhibitors increased by 25% palbociclib C_trough_ (*p* = 0.035), while antacids reduced it by 20% (*p* = 0.036). However, DDI did not have any significant effect on high-grade neutropenia occurrence (*p* > 0.05). This study confirms the major role of TDM to manage palbociclib safe use from the first week of treatment, particularly the significant incidence of hematological toxicity. Moreover, this first dedicated prospective study confirmed the importance of characterizing co-treatments to limit the DDI risk with oral-targeted therapies.

## 1. Introduction

Neutropenia is one of the most frequently reported toxicities when using oncologic drugs. Neutropenia may even be considered a biomarker of exposure for drugs targeting the cell cycle, and may be used as a surrogate marker of efficacy [1,2,3]. CKD4/6 inhibitors, such as palbociclib, are the gold standard for the treatment of metastatic breast cancer. Although these drugs cause high-grade neutropenia in almost 50% of patients, no predictive biomarker of their toxicity has been identified yet, and there is no consensus on the correlation between palbociclib exposure and neutropenia occurrence (i.e., exposure–toxicity relationship), or between the target plasma concentration and treatment efficacy (palbociclib pharmacokinetics, PK/pharmacodynamics, PD) [4,5]. PK models suggest a link between the occurrence of neutropenia and palbociclib exposure or dose [1,6,7]. Moreover, data from clinical trials indicate that a dose reduction or a prolonged pause (>7 days) decreases the toxicity grade, or even normalizes neutrophil count [8,9]. As these findings suggest a link between dose, plasma concentration, and toxicity, therapeutic drug monitoring (TDM) could be used to monitor palbociclib plasma concentration.

Practical recommendations on TDM use for targeted therapies are based on PK data, availability of analytical techniques, and clinical trials that used TDM for dose adjustments [10]. However, TDM guidelines for palbociclib are not available yet. Therefore, it is important to characterize the PK–PD–toxicity relationships of palbociclib, especially because this drug presents PK variabilities, for instance caused by drug–drug interactions (DDI) or pharmacogenetic variants [11]. Palbociclib bioavailability is moderate and pH dependent (46%) [12]. Moreover, it is largely bound to plasma protein (85.3%), leading to a significant risk of intra- and inter-individual PK variabilities.

In addition, as a substrate and inhibitor of CYP3A4, palbociclib plasmatic concentration may be modulated by co-treatments (i.e., DDI victim), but it may also lead to DDI (i.e., perpetrator). To date, only clinical cases highlighted the potential clinical relevance of these DDI (palbociclib associated with ciclosporin or verapamil), without a specific analysis of palbociclib PK [13,14]. A recent review suggested that empirical dose adjustments should be performed in function of the other drugs taken by the patient [15].

In this context, in a prospective cohort of patients with breast cancer receiving first-line palbociclib treatment, we determined palbociclib plasma concentration and evaluated its correlation with neutropenia occurrence. We also investigated the causes of PK variability, including DDI that may influence plasma palbociclib concentration.

## 2. Materials and Methods

### 2.1. Trial Design and Patients

This study used the clinical data collected in the framework of a dedicated, prospective, bicentric clinical trial to determine palbociclib exposure–toxicity correlations carried out at the Institut du Cancer de Montpellier (ICM, France) and Nîmes University Hospital (France). The trial was performed in accordance with Good Clinical Practice standards (NCT04025541). Patients with metastatic, hormone-sensitive, HER2-negative breast cancer were enrolled between June 2018 and July 2020. They all received first-line treatment with palbociclib (125 mg per day for 3–4 weeks) associated with an aromatase inhibitor. Patients were included after signature of the informed consent. After the oncology consultation and inclusion in the clinical trial, patients were interviewed by a hospital pharmacist to identify co-treatments and DDI risk, particularly CYP450 inducers or inhibitors. Treatment compliance was assessed at each visit.

### 2.2. Endpoint Analysis (Palbociclib Exposure–Toxicity Relationship)

The primary endpoint was the percentage of patients with grade 3–4 neutropenia, according to the NCI-CTCAE v4.03 criteria, during the first two palbociclib cycles, in function of its steady-state concentration (day 15 of the first cycle, D15C1). Exploratory analyses were carried out to evaluate the impact of concomitant treatments and DDI occurrence on palbociclib steady-state concentration (D15C1).

### 2.3. Pharmacokinetics

The steady-state concentration of palbociclib (plasma trough concentration; C_trough_) was quantified in all patients. For the PK analysis, blood samples were collected at D15C1 before the next dose to determine plasma concentration (C_trough_) using our previously published HPLC-MS/MS method, validated according to the Food and Drug Administration and European Medicines Agency recommendations [16]. Non-compliant patients or those whose samples were not at the residual concentration were excluded from the analysis.

### 2.4. Exposure–Toxicity Analysis

Clinical and biological toxicities were recorded at each visit, i.e., every 15 days during the first two treatment cycles. Patients were divided into two groups in function of the occurrence or not of palbociclib-induced high-grade (3–4) neutropenia during the first two treatment cycles. For each patient, the geometric median of all available palbociclib C_trough_ levels was calculated. This was compared to the geometric median value of palbociclib C_trough_ in the whole population.

### 2.5. Exposure-DDI Relationship Analysis

After the oncology consultation and inclusion in the clinical trial, patients were interviewed by a hospital pharmacist to identify co-treatments and DDI risk (part of the medication reconciliation process). Patients were classified in function of their risk of DDI that might lead to CYP3A4 and/or P-glycoprotein inhibition (P-gp) and to gastric pH increase by gastric acid-suppressive agents (e.g., proton pump inhibitors, histamine type 2-receptor blockers) using databases (e.g., DDI predictor^®^, Drugs.com^®^, PubMed^®^).

### 2.6. Statistical Analysis

Quantitative variables were described as the number of observations (N), median, interquartile range, mean and standard deviation. The Kruskal–Wallis test was used to compare the distribution of quantitative variables. Qualitative variables were described as number of observations (N) and frequency (%) of each modality. Missing values for each variable were counted. Percentages were calculated relative to the total population after exclusion of missing data. The Chi-2 test was used to compare frequencies (or the Fisher’s exact test if the expected frequencies were <5). Adjusted odds ratios (OR) and their 95% confidence interval (CI) were estimated using a logistic regression model for the occurrence of grade 3–4 neutropenia during the first two palbociclib cycles. Palbociclib C_trough_ was log-transformed and modeled using a multivariate linear regression. Multivariate model for occurrence of grade neutropenia was constructed using a backward variable selection procedure. All variables that showed a significant or moderately significant correlation (i.e., *p* < 0.20) with the primary endpoint were included as candidate variables in the initial model. Potential confounding factors were assessed at each step of the selection procedure. Functional forms of continuous variables were checked in order to assess any potential deviation from linearity in the multivariate model. All statistical tests were two-sided, and the significance level was set at 5% (i.e., *p* < 0.05). Statistical analyses were performed with STATA v16.0 and R v4.0.3.

## 3. Results

### 3.1. Patients

In total, 62 patients were included in the study between 18 June 2018 and 16 July 2020 (intention-to-treat population). However, four patients withdrew from the study before palbociclib treatment initiation (Figure S1). Among the 58 patients (*n* = 57 women; median age: 66 years), the ECOG performance status at inclusion was 0 in more than 60%. Patients were mostly menopausal (80.7%), and 67.2% of them had received at least one previous treatment at the localized stage of the disease (Table S1). Most patients (98.3%) were treated for metastatic breast cancer (except one patient with locally advanced, unresectable breast cancer), and half of them had de novo metastatic disease, with a median of one metastatic site, mainly in bone (78.9%), lymph nodes (35.1%), lung (15.8%), or liver (14%) (Table S1).

At palbociclib treatment initiation, blood count was normal in more than 80% of patients. High-grade (3–4) neutropenia was the most frequent side effect during the first two cycles of palbociclib (67.2% of patients; all grades combined: 70.7% of patients) (Table 1). Palbociclib dose was reduced by at least one dose level in 32.8% (19/58) of patients, mostly due to hematological toxicities (89.5%, 17/19 patients), and treatment was interrupted for hematological toxicity in 5.2% of patients (3/58) (Table 1). All adverse events were prospectively recorded during the follow-up, although only neutropenia was evaluated in the endpoint analysis.

### 3.2. Clinical–Biological Data and Palbociclib-Induced Toxicity

**Figure 1:**
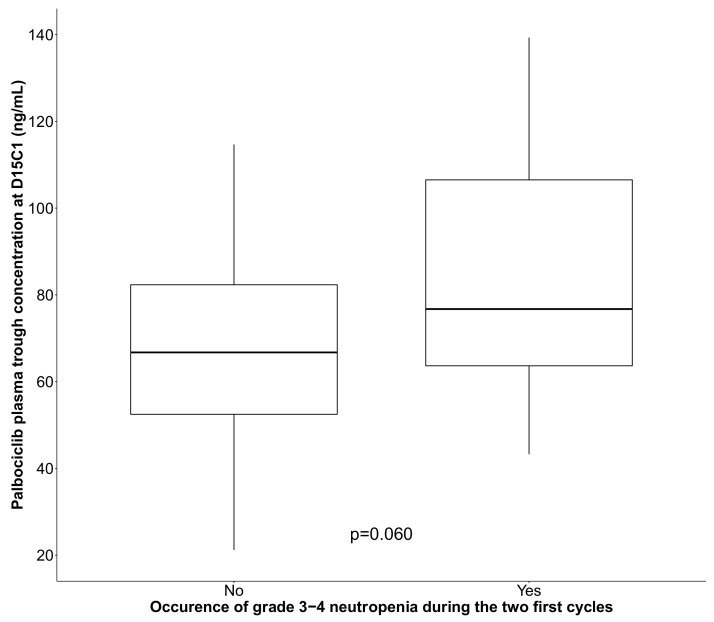
Box plot showing palbociclib plasma trough concentration at D15C1 in function of the occurrence or not of grade 3–4 neutropenia during the first two treatment cycles (black line: median). *p* value is derived from Kruskal–Wallis test.

Biological data at inclusion (blood count, kidney and liver function) were in the normal range in >80% of patients. Plasma palbociclib concentration could be quantified in 54 patients (Figure S1), and the steady-state plasma C_trough_ at D15C1 was used as an indicator for TDM. The mean ± standard deviation (range) palbociclib C_trough_ was 80.3 ± 26.7 ng/mL (21.2–130 ng/mL), and the median was 74.1 ng/mL. Median palbociclib C_trough_ (IQR) was 66.7 ng/mL (52.0–82.7) and 76.7 ng/mL (61.3–101.5) in patients without and with high grade neutropenia, respectively (Figure 1).

In univariate analysis, higher BMI (OR_increase_ 1.14, 95% CI (1.00; 1.31) *p* = 0.038), lower leukocytes (OR_increase_ = 0.61, 95% CI (0.44; 0.84)) and neutrophils (OR_increase_ = 0.62, 95% IC (0.42; 0.92)) at inclusion were significantly associated with high-grade neutropenia (Table 2). Higher palbociclib C_trough_ was also correlated with increased risk of high-grade neutropenia (OR = 1.28, 95% CI (1.01; 1.64), *p* = 0.031) (Table 2).

The final multivariate model included neutrophils count at inclusion and palbociclib C_trough_. As observed in the univariate analysis, lower neutrophils (OR_increase_ = 0.56, 95% IC (0.36; 0.86), *p* = 0.002) and higher palbociclib C_trough_ (OR_increase_ = 1.42, 95% IC (1.06; 1.90), *p* = 0.008) were significantly associated with increased risk of high-grade neutropenia (Table 2).

**Figure 2:**
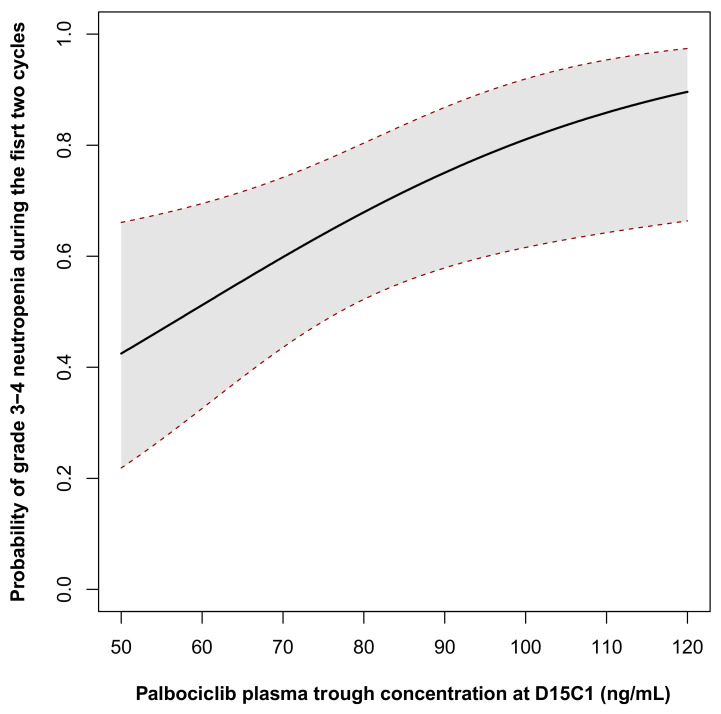
Probability of grade 3–4 neutropenia occurrence during the first two cycles in function of palbociclib trough concentration at D15C1. The probability was calculated for a patient neutrophil count at inclusion corresponded to the mean value of the cohort (62 years of age, neutrophils count = 4.3 × 10^9^/L).

From the multivariate model, a probability model of neutropenia risk in function of palbociclib C_trough_ at D15C1 was generated (Figure 2). According to this model, the probability of developing high grade neutropenia for patient with a palbociclib C_trough_ of 61, 74 and 101 ng/mL and a neutrophil count of 4.3 10^9^/L was 52% (95%CI (34%, 70%)) 63% (95% CI (47%, 76%)) and 82% (95% CI (62%, 92%)), respectively. This indicates the presence of a concentration–toxicity relationship.

### 3.3. Palbociclib Pharmacokinetics and Clinicopathological Features

In univariate analysis, plasma palbociclib C_trough_ was correlated with clinical and biological features, such as age and kidney function and albuminemia, but not with BMI.

Specifically, palbociclib C_trough_ was higher than the median concentration (74 ng/mL) in older patients (71- vs. 57-year-old, *p* = 0.002) (Table 3) and in patients with reduced kidney function (glomerular filtration rate of 80.3 vs. 93.6 mL/min, *p* = 0.017).

### 3.4. Palbociclib Exposure and Co-Medication

Among the causes of PK variability that may modulate palbociclib C_trough_, the impact of DDI (i.e., drugs taken at D15C1) was also evaluated. To this aim, the number of patients who were still taking CYP3A4 and P-glycoprotein inhibitors at D15C1 (despite the medication reconciliation at inclusion) was recorded. One third of patients (33.3%) were taking at least one CYP3A4 or P-glycoprotein inhibitor (e.g., amlodipine, nifedipine, atorvastatin, simvastatin). As palbociclib absorption is pH dependent, the influence of antacid intake was also evaluated. In our cohort, 25% of patients used antacids (proton pump inhibitors, such as pantoprazole or omeprazole, and histamine type 2-receptor blockers, such as ranitidine) at D15C1, despite the initial medication reconciliation.

**Figure 3:**
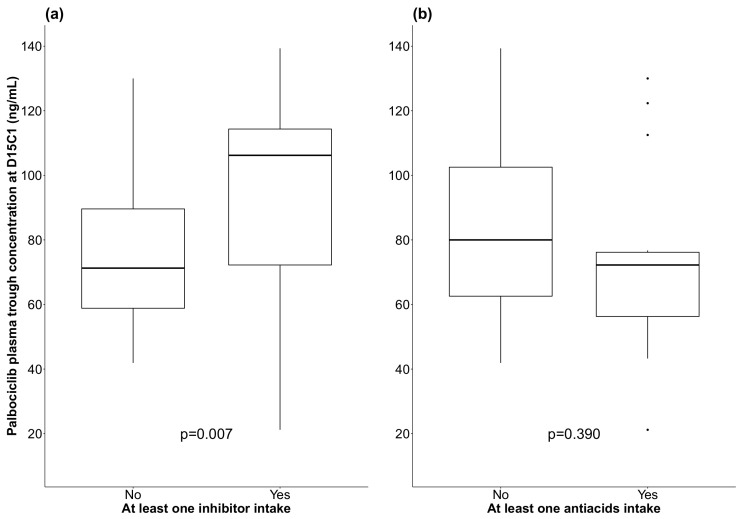
Box plots showing palbociclib plasma trough concentration at D15C1 in function of the co-intake or not of CYP3A4/P-glycoprotein inhibitors (**a**) and of antacids (**b**); (black line: median).

Palbociclib C_trough_ was higher in patients that had taken at least one CYP3A4 or P-glycoprotein inhibitor (106.1 ng/mL vs. 71.3 ng/mL, *p* = 0.007; univariate analysis, Figure 3). Median palbociclib C_trough_ was 80 ng/mL and 72.2 ng/mL for patients who took at least one antacid and those who did not, respectively (*p* = 0.390, univariate analysis, Figure 3).

To assess the impact of co-medication on palbociclib C_trough_, a multivariate analysis was carried out using a linear regression including intake of CYP3A4 or P-glycoprotein inhibitor and antacids, adjusted for age and body surface area at D15C1 (Table 4). After adjustment, the mean palbociclib C_trough_ in patients taking at least one CYP3A4 or P-glycoprotein inhibitor was significantly increased by 25% (95% CI (0.4%; 56%), *p* = 0.035) compared with patients not taking inhibitors. The mean palbociclib C_trough_ was significantly decreased by 20% in patients taking at least one antacid (95% CI (−36%; −0.3%), *p* = 0.036) compared with patients not taking them. The risk of interaction between CYP3A4 inhibitors and antacids was also tested, but it was not significant (*p* = 0.788). However, DDI was not associated with high-grade neutropenia occurrence (*p* = 0.372 for CYP3A4 inhibitors and *p* = 0.206 for antacids) (Table 3, univariate analysis).

## 4. Discussion

This prospective study investigated palbociclib exposure–toxicity relationship and PK variability in real life in 62 patients with metastatic hormone-sensitive breast cancer. The clinical–biological data were consistent with those of the PALOMA 1–3 trials: similar mean age (62.9 years vs. 60 years), but better general condition (63.8% of patients with ECOG performance status of 0 vs. 58.2% in the combined PALOMA trials) [17]. During the first two palbociclib cycles, 70.7% of patients reported neutropenia (vs. 80.6% in the combined PALOMA trials) and 67.2% high-grade neutropenia (vs. 67.1% in the PALOMA 2 and 57% in the PALOMA 1 trial) [18]. The use of dose reduction was similar (more than 30% in our study and the PALOMA trials). Conversely, treatment interruption was required for 74% of patients in the PALOMA trials but only for 20% of our patients, probably due to less stringent rules for neutrophil count thresholds in clinical settings, following the integration of the PALOMA trial data in clinical practice. In the PALOMA 2 trial, dose reduction for toxicity, mainly following high-grade neutropenia occurrence, did not result in a reduction of treatment effectiveness [19]. Palbociclib plasma C_trough_ could be estimated in 54 patients (mean: 80.3 ng/mL; median: 74.1 ng/mL). The mean C_trough_ was similar to the estimated concentration reported in the PALOMA 1 trial (88.5 ng/mL), but was higher than in the PALOMA 2 cohort (61.7 ng/mL in the Caucasian subgroup, relative to Japanese (95.4 ng/mL) and other Asians (90.1 ng/mL)) [20]. As our cohort consisted exclusively of Caucasian patients, it seems important to consider performing subgroup analyses according to the patient ethnicity.

We then tried to identify factors that may influence neutropenia occurrence. We found that the risk of developing neutropenia during the first two cycles of palbociclib was higher for patients with lower baseline neutrophil count (*p* = 0.007). This suggests that the patient’s bone marrow reserve (i.e., standard blood count) should be routinely analyzed before palbociclib initiation to characterize the risk of toxicity, because the occurrence of high-grade toxicity leads to therapeutic pauses and dose reduction. These data are comparable to the pooled analysis of the PALOMA 1 and 2 trials [21]. Although not confirmed in the multivariate analysis, higher BMI was also related to the occurrence of neutropenia, as reported in a recent study (*n* = 78) [22]. Importantly, in our patients, palbociclib was the first-line treatment for metastatic disease. Therefore, in patients receiving palbociclib as second (or more) line treatment, the risk of neutropenia could be higher because of their treatment history.

In our cohort, after adjustment, the risk of high-grade neutropenia was significantly increased with higher values of palbociclib C_trough_ (*p* = 0.008, OR_increase_ = 1.42, 95% CI (1.06; 1.90)). We also estimated the probability of high-grade neutropenia at 63% (95% CI (47%, 76%)) in patients with palbociclib C_trough_ at the median value (74 ng/mL). This prospective trial demonstrated the palbociclib pharmacokinetic–toxicity relationship and also investigated possible causes of palbociclib PK variability, thus completing a previously reported PK/PD model for palbociclib [23]. Our model estimated at 51% the risk of developing high grade neutropenia (95% CI (32%; 69%)) in patients with a palbociclib C_trough_ of ~60 ng/mL (approximately the mean value of the PALOMA clinical trials). Univariate analysis shows a higher palbociclib concentration in patients with lower renal clearance, despite the low proportion of palbociclib elimination by the renal route (17%). A clinical study showed that in patients with impaired renal function, palbociclib plasma concentration is higher, but can be used safely in this population [24]. DDI impact on palbociclib-induced neutropenia was also assessed, based on concomitant treatments at D15C1. The use of CYP3A4 or P-glycoprotein inhibitors and antacids influences palbociclib plasma concentration significantly. Palbociclib concentration was increased (+26%) when combined with at least one CYP3A4 or P-glycoprotein inhibitor (*p* < 0.05; multivariate analysis). The influence of such inhibitors on palbociclib PK has been increasingly characterized, for instance for erythromycin, a moderate CYP3A4 inhibitor (*n* = 11) [25]. Although the drugs involved in our analysis are not described as major inhibitors (simvastatin, atorvastatin, amlodipine, losartan or nifedipine), their influence was found to be statistically significant. Conversely, we observed a significant reduction in palbociclib C_trough_ concentration (20%, *p* < 0.05) in patients taking antacids at D15C1. However, we did not find any correlation between these DDI and the occurrence of neutropenia (*p* = 0.239). This can certainly be explained by the small size of our cohort (*n* = 62). The impact of these co-treatments in terms of survival will be evaluated later, especially because the correlation between palbociclib plasma concentration and hematologic toxicity can lead to dose reductions. Recent studies suggest a link between co-medication (statin use) and neutropenia occurrence (*n* = 78), and a negative influence of antacids on the survival of patients treated with palbociclib (*p* < 0.0001) [22,26]. It would be relevant to analyze the various PK/PD correlations and specifically the modulation of palbociclib concentration on treatment efficacy in our cohort. However, we could not investigate this point because the survival data of our cohort are not available yet. The clinical impact of DDI is becoming better characterized, for instance the negative influence of antacid use on survival in patients with sarcoma treated with pazopanib [27]. Although target concentrations are not yet clearly defined for palbociclib, TDM appears to be a relevant tool for improving patient management, especially in view of the frequent occurrence of hematological toxicity. TDM is also a way to characterize and estimate the relevance of the causes of PK variability.

## 5. Conclusions

The pharmacokinetic–toxicity relationship and PK variability of palbociclib were characterized in real-life metastatic breast cancer patients (*n* = 62). The risk of high-grade neutropenia was significantly associated with higher values of palbociclib C_trough_ (*p* = 0.008, OR_increase_ = 1.42, 95% CI (1.06; 1.90)). Cotreatment, as CYP3A4 or P-glycoprotein inhibitors or antacids, were significantly modulated palbociclib C_trough_ (+/−20%). Clinical pharmacy activity and TDM allows characterization of DDI risk and ensures safety and efficacy of the CDK4/6 inhibitor as palbociclib.

## Figures and Tables

**Table 1:** Treatment interruption/dose modification and toxicity occurrence (safety population, *n* = 58).

Number of Palbociclib Cycles (*n*)	
*n*	58
Mean (SD)	8.9 (3.6)
Median (Q1;Q3)	10.5 (6.0; 12.0)
**Duration of treatment (months)**	
*n*	58
Mean (SD)	8.9 (3.8)
Median (Q1;Q3)	11.0 (6.4; 11.3)
**Dose reduction (*n*)**	
At least one dose reduction	
No	39 (67.2%)
Yes	19 (32.8%)
If yes:	
For hematologic toxicity	17 (89.5%)
For other toxicity	2 (10.5%)
**Treatment interruption (*n*)**	
At least one treatment interruption	
No	46 (79.3%)
Yes	12 (20.7%)
If yes:	
For hematologic toxicity	3 (25.0%)
For other toxicity	9 (75.0%)
**Neutropenia during the first two cycles (*n*)**	
Grade during the first two cycles	
Grade 0	17 (29.3%)
Grade 1	1 (1.7%)
Grade 2	1 (1.7%)
Grade 3	34 (58.7%)
Grade 4	5 (8.6%)
Incidence of grade 3+ neutropenia during the first two cycles	
No	19 (32.8%)
Yes	39 (67.2%)

**Table 2:** Univariable and multivariable analysis for occurrence of grade 3–4 neutropenia during the first two palbociclib cycle. Patients evaluable for safety (*n* = 58).

Variable	Nb Evt/N	Univariable Analysis			Multivariable Analysis		
		OR	95% IC	*p* Value ^‡^	OR	95% IC	*p* Value ^‡^
Clinical variables							
Age				*p* = 0.928			
5 years increase	39/58	0.99	(0.80; 1.22)				
BMI (kg/m²)				*p* = 0.038			
1-unit increase	37/56	1.14	(1.00; 1.31)				
Missing	2						
Previous treatment				*p* = 0.461			
No	14/19	1.00	Ref				
Yes	25/39	0.64	(0.19; 2.14)				
Laboratory data							
Lymphocytes (10^9^/L)				*p* = 0.101			
1-unit increase	39/58	0.64	(0.37; 1.12)				
Leukocytes (10^9^/L)				*p* = 0.001			
1-unit increase	39/58	0.61	(0.44; 0.84)				
Neutrophils (10^9^/L)				*p* = 0.007			*p* = 0.002
1-unit increase	39/58	0.62	(0.42; 0.92)		0.56	(0.36; 0.86)	
Hemoglobin (g/dL)				*p* = 0.103			
1-unit increase	39/57	1.43	(0.92; 2.25)				
Bilirubin (g/dL)				*p* = 0.201			
1-unit increase	36/55	1.11	(0.94; 1.31)				
Kidney clearance (mL/min/1.73 m²)				*p* = 0.538			
10-unit increase	39/58	0.92	(0.71; 1.19)				
Treatment data at D15C1							
Palbociclib C_trough_				*p* = 0.031			*p* = 0.008
10 unit increase	35/54	1.28	(1.01; 1.64)		1.42	(1.06; 1.90)	
CYP3A4 and/or p-gp inhibitor				*p* = 0.318			
No	14/19	1.00	Ref				
Yes	25/39	0.55	(0.17; 1.77)				
Antacids				*p* = 0.183			
No	28/40	1.00	Ref				
Yes	7/14	0.43	(0.12; 1.49)				

^‡^ Log-likelihood ratio test.

**Table 3:** Univariate analysis of the correlation between plasma trough concentration of palbociclib at D15C1 and selected variables. Patients evaluable for safety with usable Ctrough data (*n* = 54).

	Palbociclib C_trough_		All	Test
	≤74 ng/mL	>74 ng/mL		
	*n* = 27	*n* = 27	*n* = 54	
**Sociodemographic and clinical variables at inclusion**				
Age (years)				*p* = 0.002
N	27	27	54	
Mean (SD)	57.1 (12.9)	67.8 (12.3)	62.5 (13.6)	
Median (Q1;Q3)	57.0 (48.0; 67.0)	71.0 (64.0; 76.0)	65.5 (55.0; 74.0)	
Age (Median)				*p* = 0.003
≤66 years	20 (74.1%)	9 (33.3%)	29 (53.7%)	
>66 years	7 (25.9%)	18 (66.7%)	25 (46.3%)	
BMI (kg/m²)				*p* = 0.963
N	25	27	52	
Mean (SD)	25.5 (4.9)	25.5 (4.5)	25.5 (4.7)	
Median (Q1;Q3)	25.4 (22.1; 29.0)	24.6 (22.5; 28.1)	25.0 (22.2; 28.5)	
Missing	2	0	2	
Alcohol consumption				*p* = 1.000
Non consumer	23 (85.2%)	23 (85.2%)	46 (85.2%)	
Former consumer	0 (0.0%)	1 (3.7%)	1 (1.9%)	
Consumer	4 (14.8%)	3 (11.1%)	7 (13.0%)	
Tobacco consumption				*p* = 0.322
Non-smoker	17 (63.0%)	22 (81.5%)	39 (72.2%)	
Former smoker	5 (18.5%)	2 (7.4%)	7 (13.0%)	
Smoker	5 (18.5%)	3 (11.1%)	8 (14.8%)	
**Biological variables at inclusion**				
Creatinine (μmol/L)				*p* = 0.166
N	27	27	54	
Mean (SD)	66.9 (15.9)	70.1 (13.4)	68.5 (14.7)	
Median (Q1;Q3)	63.0 (58.0; 70.0)	66.0 (62.3; 79.0)	64.5 (59.0; 74.3)	
Kidney clearance (ml/min/1.73 m^2^)				*p* = 0.017
N	27	27	54	
Mean (SD)	93.6 (24.2)	80.3 (18.2)	87.0 (22.3)	
Median (Q1;Q3)	96.0 (87.0; 103.0)	81.0 (67.0; 96.0)	88.5 (70.0; 100.0)	
Albumin (g/L)				*p* = 0.040
N	21	25	46	
Mean (SD)	43.6 (4.5)	41.3 (4.1)	42.3 (4.6)	
Median (Q1;Q3)	43.0 (41.7; 47.0)	42.0 (39.0; 43.5)	42.0 (40.0; 45.0)	
Missing	6	2	8	

**Table 4:** Adjusted association between comedications at D15C1 and log-concentration at D15C. Multivariate linear regression (*n* = 52).

Variable	*n* = 52	
	Coefficient	95% IC
CYP3A4/P-gp inhibitors		*p* = 0.035
No	1.00	Ref
Yes	0.22	(0.01; 0.44)
Antacids		*p* = 0.036
No	1.00	Ref
Yes	−0.23	(−0.46; −0.01)
Body surface area at D15C1		*p* = 0.787
0.5 m² increase	−0.03	(−0.31; 0.24)
Age		*p* = 0.146
5 years increase	0.03	(−0.01; 0.06)

## Data Availability

Data are available on request due to restrictions, e.g., privacy or ethical. The data presented in this study are available on request from the corresponding author.

## Supplementary Materials

The following supporting information can be downloaded at: https://www.mdpi.com/article/10.3390/pharmaceutics14040841/s1, Figure S1: Study flow chart; Table S1: Socio-demographic and tumor anatomopathological characteristics at inclusion. Population evaluable for toxicity (*n* = 58).
