# Supplementary material for: Pharmacokinetic Variability Drives Palbociclib-Induced Neutropenia in Metastatic Breast Cancer Patients: Drug–Drug Interactions Are the Usual Suspects
Source: Pharmaceutics. 2022 Apr 11;14(4):841. doi: 10.3390/pharmaceutics14040841 (PMC9032884; doi:10.3390/pharmaceutics14040841)
Supplement: Supplementary file 1 [file pharmaceutics-14-00841-s001.zip › pharmaceutics-1650008-supplementary.pdf]

pharmaceutics

Supplementary Materials: Pharmacokinetic Variability Drives Palbociclib-Induced Neutropenia in Metastatic Breast Cancer Patients: Drug–Drug Interactions Are the Usual Suspects

Fanny Leenhardt, Frédéric Fiteni, Ludovic Gauthier, Marie Alexandre, Séverine Guiu, Nelly Firmin, Stéphane Pouderoux, Marie Viala, Gerald Lossaint, Chloé Gautier, Caroline Mollevi, Matthieu Gracia, Celine Gongora, Litaty Mbatchi, Alexandre Evrard and William Jacot

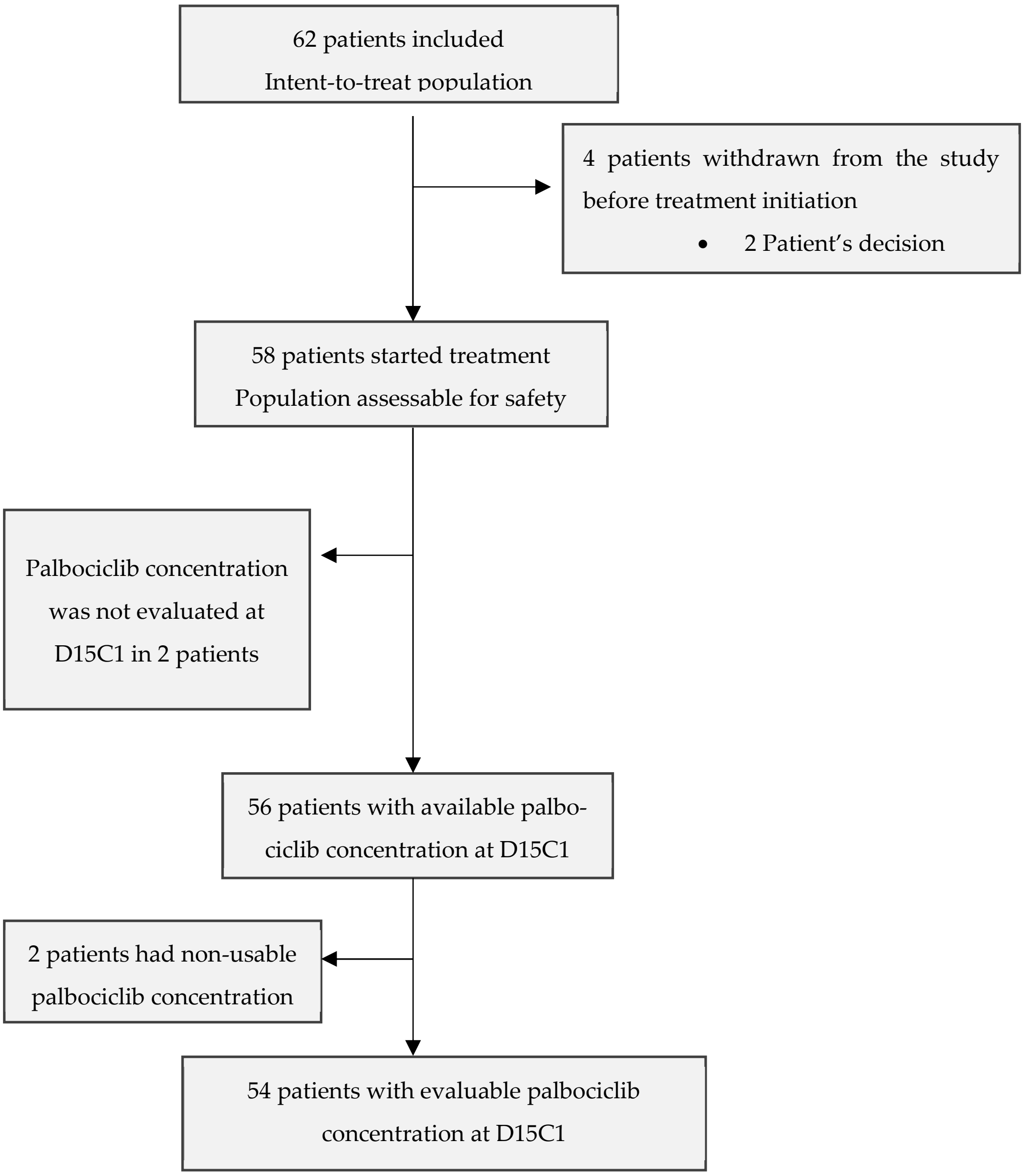

Figure S1. Study flow chart.

Table S1. Socio-demographic and tumor anatomopathological characteristics at inclusion. Population evaluable for toxicity (*n* = 58).

**Age (years)**	
Mean (SD)	62.9 (13.2)
Median (Q1;Q3)	66.0 (55.0; 74.0)
**Ethnic origin**	
Caucasian	58 (100%)
**Sex**	
Man	1 (1.7%)
Woman	57 (98.3%)
**If woman:**	
**Menopausal status**	
No	11 (19.3%)
Yes	46 (80.7%)
**Weight at inclusion (kg)**	
Mean (SD)	68.0 (13.7)
Median (Q1;Q3)	66.0 (57.0; 77.0)
**BMI (kg/m²) (n=56)**	
Mean (SD)	25.7 (4.8)
Median (Q1;Q3)	25.0 (22.3; 28.5)
Missing	2
**ECOG Performance Status at inclusion**	
0	37 (63.8%)
1	15 (25.9%)
2	6 (10.3%)
**Previous treatment (early stage)**	
No	19 (32.8%)
Yes	39 (67.2%)
**If yes (*n* = 39):**	
Radiotherapy	30 (76.9%)
Chemotherapy	25 (64.1%)
Hormone therapy	16 (41.0%)
Surgery	35 (92.1%)
**If metastatic breast cancer (*n* = 57)**	
**Number of metastatic sites**	
Mean (SD)	1.6 (0.9)
Median (Q1;Q3)	1.0 (1.0; 2.0)
**De novo metastasis**	
No	29 (50.9%)
Yes	28 (49.1%)
**Metastasis sites**	
Nodes	20 (35.1%)
Liver	8 (14.0%)
Lymph nodes	20 (35.1%)
Lung	9 (15.8%)
Pleura	5 (8.8%)
Bone	45 (78.9%)

Peritoneum	2 (3.5%)
Others (like skin, uterus, …)	3 (5.2%)
**Estrogen receptor expression**	
Negative	1 (1.7%)
Positive	57 (98.3%)
**Progesterone receptor expression**	
Negative	7 (12.1%)
Positive	51 (87.9%)
**HER2 status**	
Negative	55 (94.8%)
Positive	3 (5.2%)
